# Quantitative Electroencephalographic Biomarkers in Preclinical and Human Studies of Huntington’s Disease: Are They Fit-for-Purpose for Treatment Development?

**DOI:** 10.3389/fneur.2017.00091

**Published:** 2017-03-30

**Authors:** Michael K. Leuchter, Elissa J. Donzis, Carlos Cepeda, Aimee M. Hunter, Ana María Estrada-Sánchez, Ian A. Cook, Michael S. Levine, Andrew F. Leuchter

**Affiliations:** ^1^David Geffen School of Medicine, University of California Los Angeles (UCLA), Los Angeles, CA, USA; ^2^Intellectual and Developmental Disabilities Research Center, David Geffen School of Medicine, Semel Institute for Neuroscience and Human Behavior, University of California Los Angeles (UCLA), Los Angeles, CA, USA; ^3^Department of Psychiatry and Biobehavioral Sciences, David Geffen School of Medicine, University of California Los Angeles (UCLA), Los Angeles, CA, USA; ^4^Neuromodulation Division, Laboratory of Brain, Behavior, and Pharmacology, Semel Institute for Neuroscience and Human Behavior, University of California Los Angeles (UCLA), Los Angeles, CA, USA; ^5^Department of Bioengineering, University of California Los Angeles (UCLA), Los Angeles, CA, USA

**Keywords:** Huntington’s disease, mutant huntingtin, huntingtin aggregates, quantitative electroencephalography, biomarkers, human, mouse, electrophysiology

## Abstract

A major focus in development of novel therapies for Huntington’s disease (HD) is identification of treatments that reduce the burden of mutant *huntingtin* (mHTT) protein in the brain. In order to identify and test the efficacy of such therapies, it is essential to have biomarkers that are sensitive to the effects of mHTT on brain function to determine whether the intervention has been effective at preventing toxicity in target brain systems before onset of clinical symptoms. Ideally, such biomarkers should have a plausible physiologic basis for detecting the effects of mHTT, be measureable both in preclinical models and human studies, be practical to measure serially in clinical trials, and be reliably measurable in HD gene expansion carriers (HDGECs), among other features. Quantitative electroencephalography (qEEG) fulfills many of these basic criteria of a “fit-for-purpose” biomarker. qEEG measures brain oscillatory activity that is regulated by the brain structures that are affected by mHTT in premanifest and early symptom individuals. The technology is practical to implement in the laboratory and is well tolerated by humans in clinical trials. The biomarkers are measureable across animal models and humans, with findings that appear to be detectable in HDGECs and translate across species. We review here the literature on recent developments in both preclinical and human studies of the use of qEEG biomarkers in HD, and the evidence for their usefulness as biomarkers to help guide development of novel mHTT lowering treatments.

## Introduction

Many current strategies for the treatment of Huntington’s disease (HD) are focused on development of agents to lower mutant huntingtin (mHTT) protein burden. Although the precise role of mHTT aggregates in HD neuropathology has not been completely elucidated, studies in HD transgenic mice have shown a correlation between mHTT aggregation and development of the phenotype (1). The presence of mHTT adversely affects the function of multiple cell lines, particularly neurons, from early human development through phenoconversion to the illness. Therefore, chronic reduction in the levels of mHTT prior to the onset of symptoms could presumably prevent the development or reduce the severity of many of the manifestations of HD.

Biomarkers play a unique role in research aimed at mHTT reduction. In contrast to most degenerative diseases of the central nervous system, highly specific biomarkers for HD already exist: the genetic mutation that leads to production of mHTT (a trinucleotide repeat expansion) has been identified. It is already possible to identify individuals who will develop the illness based on the presence of the mutation, estimate their age of disease onset (by measuring length of the expansion), and to quantify levels of mHTT in a subject’s cerebrospinal fluid and body tissues.

Although these biomarkers are highly specific, they are not entirely fit-for-purpose for the development of mHTT lowering therapeutic strategies. While mHTT is produced throughout life, the developmental stage at which the protein becomes toxic varies across organ systems, and even within a particular organ. mHTT toxicity is remarkably pleiotropic (2, 3), disrupting a broad range of cellular processes including energetics (i.e., mitochondrial function), transcriptional regulation (i.e., expression of neurotrophic factors and G protein-coupled receptors), synaptic neurotransmission (i.e., vesicular trafficking), and synaptic plasticity (2, 4–6). It remains unclear at what stage of development mHTT begins to elicit these different forms of toxicity and whether different brain regions (i.e., cortex, striatum, and thalamus) have differential sensitivities to mHTT that may change over the course of nervous system development. Therefore, while it is feasible to measure mHTT levels, it is difficult to use mHTT levels alone to gauge the efficacy of mHTT lowering strategies in achieving clinically meaningful outcomes.

The Huntingtin-Lowering Biomarker Task Force (7) posited nine criteria for biomarkers of sufficient reliability to be utilized in trials of mHTT lowering agents. They concluded that such biomarkers should share several characteristics: (1) biological plausibility (a plausible physiologic connection between biomarker readout and change in mHTT level); (2) technologic feasibility (adaptable to both humans and animal models); (3) measurable in humans (useful in humans participating in clinical trials); (4) repeatable within subjects (good test–retest reliability with serial use during treatment); (5) reliably measurable in HD gene expansion carriers (HDGECs) (applicable to pre-manifest and early- to mid-stage manifest HD); (6) signal metrics (good signal-to-noise ratio and dynamic range, applicable to relatively small sample sizes, and strong inter- and intra-subject and site reliability); and (7) measurable in preclinical HD models (quantifiable in HD animal models).

Quantitative electroencephalography (qEEG) biomarkers fulfill many of the criteria outlined above for use in research and clinical trials to develop mHTT lowering treatments. qEEG biomarkers have a plausible physiologic link to the effects of mHTT lowering because brain oscillatory activity is regulated by the thalamocorticostriatal systems that show preclinical and early-disease changes in HD. The technology is feasible to implement across species and can easily be performed serially in the course of clinical trials. A key question is whether reliable and reproducible biomarker signals can be measured in preclinical and clinical studies involving HD and HDGECs. We review here the animal and human literature on qEEG biomarkers and discuss the role that these biomarkers may fulfill in trials aimed at development of mHTT lowering treatments.

## Methods

We performed searches on PubMed for the years 2000–2016 aimed at identification of all studies that examined qEEG biomarker features in preclinical and clinical models of HD. All searches in the series included (EEG OR EEG Mapping OR qEEG) AND (Huntington’s disease OR Huntington’s OR huntington OR huntington disease). Additional query terms for individual searches included biomarker, preclinical, power spectral analysis, neural networks, LORETA, (Mouse OR Mice) AND (R6/2 OR zQ175), (monkey OR non-human primate), rat, sheep, minipig, and animal model. Single-subject case studies and publications that met PubMed search criteria but did not discuss EEG findings were excluded from our results. A total of 37 entries were preliminarily identified, of which 21 met our criteria for inclusion in this review. Further elimination of duplicated entries from different searches yielded 16 unique publications for review.

## Results

### Preclinical Studies

#### Rodent Models

The most common models of HD utilized in preclinical studies involve transgenic mice, particularly R6/2 and zQ175 (8, 9). These models allow for the relatively rapid evaluation of biomarkers from shortly after birth to symptom onset and recapitulate characteristics of the disease seen in humans (9, 10). The R6/2 mouse carries a fragment of exon 1 of the human mHTT gene with an expanded CAG-repeat segment that shows relatively early onset of rapidly progressive symptoms (10, 11). The original R6/2 mice had a CAG repeat length of approximately 150, and a lifespan of roughly 15 weeks, with onset of symptoms at approximately 6–7 weeks of age. Contrary to expectation, increasing the CAG repeat length in R6/2 mice led to a lessening of symptom severity and longer lifespan. Current studies frequently utilize R6/2 mice with a CAG repeat length of 250 (12). The zQ175 knock-in (KI) mouse model, which expresses the mHTT gene within the mouse genomic context (13), allows for further examination over the course of a slower progression that more closely follows the course of human disease (13–16). The resulting neural circuitry disruptions are observable through qEEG.

Table 1 shows results of the PubMed search of articles discussing qEEG in R6/2 and zQ175 mice, as well as tgHD and BACHD rats. Five studies examined qEEG findings in R6/2 mice, comparing qEEG recorded from cortical or dural electrodes with those seen in wild-type (WT) mice across the sleep–wake cycle. Fisher et al. (17) characterized sleep qEEG findings from dural screw electrodes following periods of sleep deprivation and found both a progressive reduction of sleep time and fragmentation of sleep in R6/2 mice when compared to WT mice (17). These progressive changes were observable as early as 13 weeks of age (17). Similar findings were reported by Kantor et al. (18), although this group detected differences from WT by 9 weeks of age (18). In addition to diurnal patterns, Fisher and colleagues examined the circadian measure of body temperature and found that R6/2 mice also exhibited disruption of this regulation by 13 weeks (17).

**Table 1:** **Literature review results for qEEG biomarkers in rodents**.

Reference	Year	Rodent strain(s)	Recording site	Measure	Electrophysiological finding(s)	Behavioral correlation(s)
Kantor et al. (19)	2016	R6/2 mice	Dural screws	qEEG	Gamma absolute power was higher in R6/2 mice during REM and suppressed upon administration of zolpidem or amitriptyline (*p* < 0.01)	Sleep/wake abnormalities were corrected in a dose-dependent manner and correlated with qEEG corrections
					Absolute delta power was increased by zolpidem in wild-type (WT) mice, not in R6/2, during REM (*p* < 0.01)	
					1–5 Hz (delta/alpha) and 3–7 Hz (delta/alpha/theta) absolute powers were decreased by amitriptyline in WT and R6/2, respectively (*p* < 0.01)	

Fisher et al. (16)	2016	zQ175 mice	Dural screws	qEEG	Absolute delta power lower in Het and Hom zQ175 relative to WT (*p* < 0.01)	Sleep/wake amount and body temperature regulation disrupted in Hom, qEEG markers were observed prior to disruptions. No differences observed between Het and WT, though qEEG differences observed in Het were observed prior to previously reported motor disturbances
					Absolute beta and gamma power higher in Het and Hom zQ175 relative to WT (*p* < 0.01)	
					Peak theta frequency was lower in Het and Hom zQ175 relative to WT (*p* < 0.05)	

Callahan and Abercrombie (20)	2015	R6/2 mice	Cortex	ECoG and local field potentials (LFPs) (of STN)	Absolute delta and theta power lower in R6/2 mice relative to WT (*p* < 0.01)	None
					Absolute beta (*p* < 0.01) and gamma (*p* < 0.05) power higher in R6/2 mice relative to WT	

Nagy et al. (14)	2015	zQ175 mice and BACHD rats	Hippocampus	LFP	Absolute gamma power higher in Hom zQ175 relative to WT (*p* < 0.05)	None
					Absolute gamma power lower in BACHD rats relative to WT (*p* < 0.05)	
					PDE9A inhibitor lowered WT rat absolute gamma power to BACHD levels (*p* < 0.05)	
					PDE9A inhibitor had no effect on zQ175 relative to WT (*p* < 0.05)	

Kantor et al. (18)	2013	R6/2 mice	Dural screws	qEEG	Absolute delta power lower in R6/2 mice in frontoparietal and frontal regions relative to WT (*p* < 0.05)	EEG markers were detected prior to any symptoms
					Absolute theta and delta power lower in R6/2 mice in frontal region relative to WT (*p* < 0.05)	
					Absolute gamma power higher in R6/2 mice relative to WT (*p* < 0.01)	
					Peak theta frequency shifted from 7 to 6 Hz in R6/2 mice relative to WT (*p* < 0.01)	

Fisher et al. (17)	2013	R6/2 mice	Dural screws	qEEG	High-frequency beta and gamma (25–60 Hz) absolute power higher in R6/2 during wake relative to WT (*p* < 0.05)	Circadian rhythm disturbances were found (body temperature, sleep/wake disturbances) in R6/2 mice relative to WT, but EEG abnormalities were detected earlier
					Absolute delta and theta power lower in R6/2 during NREM relative to WT (*p* < 0.05)	

Miller et al. (21)	2011	R6/2 mice	Striatum	LFP	Delta and theta relative power lower in R6/2 relative to WT (*p* < 0.05)	None
					Gamma relative power higher in R6/2 relative to WT (*p* < 0.05)	

*Base search terms included (EEG OR EEG Mapping OR qEEG) AND (Huntington’s Disease OR Huntington’s OR Huntington OR Huntington Disease). Additional terms included biomarker, preclinical, power spectral analysis, neural networks, low-resolution electromagnetic tomography (LORETA) (Mouse OR Mice) AND (R6/2 OR zQ175), and rat. Results were restricted to rodents. Information noted includes total number of species examined, the electrode recording site and recording measure used, any relevant qEEG-related findings, and correlations of those findings to behavioral and genetic measures*.

In both of these studies, circadian disruptions were accompanied by increases in high-frequency oscillatory activity (high-beta and gamma) and decreases in delta and theta absolute power in the week nine qEEG recordings (17, 18). Fisher and colleagues (17) reported that the qEEG alterations emerged at 9 weeks (4 weeks earlier than the diurnal disturbances) (17), while Kantor’s group observed qEEG differences simultaneously with diurnal disturbances (18). Because both of these groups of investigators performed their first qEEG recordings at 9 weeks of age, it is unknown whether any qEEG or diurnal disturbances would be observable prior to this age. The detection of qEEG differences prior to or in the earliest stages of symptoms suggests that qEEG differences may be detectable in HDGECs (17, 18).

Callahan and Abercrombie (20) examined the oscillatory signals arising both in the subthalamic nucleus (STN) and the cortex in anesthetized R6/2 mice using local field potentials (LFPs) and cortical recordings (20). They saw a decrease in absolute delta power and an increase in absolute beta and gamma power, corroborating previous findings. These investigators also reported reduced coherence between STN and cortex in the delta frequency band during synchronized, though not desynchronized cortical activity. Previous examination of LFPs in R6/2 mice showed high-frequency (32 Hz) oscillations during rest and grooming, while WT mice did not. Interestingly, when R6/2 mice engaged in exploration, LFPs synchrony between motor cortex and dorsal striatum declined, particularly at high frequencies (22).

Kantor and colleagues (19) performed a follow-up study of their previously observed qEEG and behavioral changes in R6/2 mice following the administration of hypnotic agents (19). Aberrant gamma oscillations were dampened by both amitriptyline and zolpidem administration in a dose-dependent manner. They also found that the abnormally high amount of REM sleep previously noted by both Kantor et al. (18) and Fisher et al. (17) was reduced. Zolpidem administration reduced the amount of REM sleep in R6/2 mice for 2–3 h, but had no effect on WT mice. Amitriptyline reduced the amount of REM sleep in both R6/2 and WT mice for at least 6 h. Amitriptyline reduced low frequency oscillatory power during NREM sleep in both WT and R6/2 mice, in addition to lengthening and consolidating NREM sleep. Zolpidem, however, induced an increase in absolute delta power and altered NREM sleep only in WT mice, not in the R6/2 model. These findings suggest a pharmacodynamic difference that may be related to the HD genotype.

Fisher and colleagues (16) performed a follow-up to their earlier R6/2 study in which they examined both heterozygous (Het) and homozygous (Hom) zQ175 mouse models (16). Findings in the Hom zQ175 model were similar to those reported earlier in R6/2 mice although with later onset of diurnal disturbances (24 vs. 13 weeks) and core temperature regulation (16 vs. 13 weeks). Diurnal and body core temperature discrepancies were not observed between Het and WT mice, though Het mice had significantly lower weight than WT mice by 29 weeks. qEEG differences were observed in both Het and Hom mice relative to WT. In both Het and Hom, absolute delta power decreased, absolute beta and gamma power increased, and peak theta frequency shifted lower relative to WT. These described spectral shifts were greater for the Hom mice than the Het. The conservation of these observations in different mouse lines suggests that they are characteristics of the disease that are expressed across different transgenic models, which is encouraging for identification of biomarkers that are sensitive to the underlying mechanisms of cell pathology across models. Additionally, the gene dosage dependence of the qEEG power suggests a potential correlation between the magnitude of the disruption of normal oscillatory patterns and severity of the underlying pathology, a desirable characteristic of the biomarker.

However, the zQ175 Het model in this study showed no symptoms of disease onset, motor or otherwise, beyond the change in body weight (16). The EEG disturbances observed in the Het mice suggest HD pathology, but the link to phenoconversion remains unclear. Previous work has demonstrated that Het mice develop behavioral symptoms, and reason for the lack of apparent symptoms in this particular study is unknown (13, 15, 23). Further research is needed to establish a closer linkage between the symptoms and qEEG biomarkers in the zQ175 Het model. Such longitudinal research may provide valuable insight into the alterations of qEEG biomarkers as a function of disease severity.

Rat models of HD have been examined in addition to the murine models described earlier, although relatively little qEEG work has been performed in rats at this time (14, 21, 24). Miller et al. (21) performed extracellular recordings of transgenic mice and rats (21). Specifically, they examined neuronal spike activity in both R6/1 and R6/2 mice, as well as KI mice and transgenic (tgHD) rats. Recordings were performed in the striatum in all models, the prefrontal cortex and primary motor cortex of R6/2 mice, and the prefrontal cortex of KI mice. LFPs also were measured in the R6/2 striatum. Significant differences in spiking activity were detected between all models and their respective WT littermates. These differences were seen in burst firing declines, inter-spike-interval variation coefficient reduction, and of significant note to this review, a decrease in delta and theta relative power and increase in relative gamma power in LFP recordings from R6/2 striata during rest.

Nagy et al. (14) examined hippocampal and auditory cortex absolute gamma power using LFPs in both zQ175 mice and BACHD rats to measure auditory gating (14). They found impairment of auditory gating in Het and Hom zQ175 mice as well as BACHD rats. They also found elevated hippocampal gamma power in Hom zQ175 mice relative to WT and decreased hippocampal absolute gamma power in BACHD rats relative to WT. However, they were able to improve auditory gating in BACHD rats (but not Het or Hom zQ175 mice) to the point of eliminating statistically significant differences between the BACHD rats and the WT through administration of a phosphodiesterase 9A inhibitor. Interestingly, absolute gamma power in WT rats also was decreased by this inhibitor, eliminating statistical differences between the BACHD and WT rats. This finding indicates pharmacologic activity in the WT rats of unclear significance. The treatment had no effect on auditory gating in the WT rats and absolute gamma power in BACHD rats was unaffected by the treatment. The relevance of these pharmacologic effects to research on mHTT lowering compounds is unclear because none of these interventions involved administration of an agent that is known to be disease modifying. Nevertheless, the findings demonstrate that pharmacologic interventions may have distinct effects in different preclinical models.

A number of potential biomarkers are reported with some consistency across studies. Five of the seven studies in Table 1 examined the relative differences across all broad frequency bands in HD mice. Kantor et al. (19) focused on the effects of hypnotic agents on these already-established differences, while Nagy et al. focused on absolute gamma power in the context of a phosphodiesterase inhibitor. The five remaining studies reported a decrease in delta power (absolute power in four of the five studies), regardless of the specific mouse model used. Six studies (Nagy included) that examined gamma power in mice found a higher gamma power in HD mice relative to WT, regardless of strain used (absolute power in five of the six studies). Theta power was found to be lower in HD models relative to controls in four of five mouse studies (absolute power in three of four studies). Absolute beta power was found to be higher in HD models relative to controls in three of five mouse studies.

These rodent studies demonstrate that qEEG biomarkers preceded behavioral symptoms in most cases, even when such disturbances were minimal (14, 16–21, 24). The only exception to this was Kantor et al. (18), where the qEEG biomarkers and some behavioral disturbances were concurrently observed during the first recordings at 9 weeks of age; it is unknown if qEEG biomarkers were present prior to the early behavioral symptoms (18). Additionally, the studies indicate that findings from rodent preclinical models are readily reproducible, quantifiable, and subject to low variability (25–27). While murine models serve as an excellent proof-of-concept, they may not accurately reflect the distribution of metabolites throughout the brain as seen in humans due to both the size of the rodent and large anatomical discrepancies between a human and mouse brain (8–10, 28). Additionally, while a maximum lifespan of roughly 36 months allows for observation of the full disease course in a feasible manner, rodent models may not accurately recapitulate the rate of disease progression seen in humans (9–11).

#### Sheep Models

The OVT73 transgenic sheep is a model of emerging interest to examine mHTT lowering. This model expresses a complete copy of human Htt with a polyglutamine expansion 73 repeats in length (9, 29). The model may be particularly useful to evaluate the safety, brain biodistribution, and effects of the expanded mHTT gene in a large brain. Additionally, recent data suggest that the sheep HD model may be helpful in assessing the effects of emerging therapies on mHTT aggregation, receptor status in striatum, and early behavioral deficits (10). A literature search for the OVT73 model indicates that no qEEG results have been published in the OVT73 sheep at the time of this review. qEEG has been examined, however, in the CLN5 Batten disease sheep model (30). Perentos et al. (30) demonstrated that qEEG can be used longitudinally in ovine models to characterize brain function as well as sleep patterns. They reported that the motor activity of rumination is affected in the CLN5 sheep relative to WT. This finding was confirmed by Nicol and colleagues (31), indicating that rumination can be used as a measure of motor dysfunction (31). Both studies demonstrated the feasibility of recording qEEG, studying sleep, and examining motor function, supporting the use of qEEG in OVT73 HD model. Further characterization of this model may be in order, however, prior to initiation of qEEG studies. While Reid et al. (32) have demonstrated striatal transcripts indicative of gene expression (32, 33) and Handley et al. (33) have demonstrated altered brain metabolic activity in 5-year-old OVT73 sheep (33), Morton et al. (34) showed that the OVT73 sheep exhibit no structural differences using MRI relative to the WT sheep, only circadian differences (34), when studied until 5 years of age. The lack of onset of obvious brain structural changes or motor symptoms suggests the presentation of HD in OVT73 may be less severe or incomplete. However, the development of qEEG and rumination recording methods in the CLN5 sheep supports the usefulness of ovine research and supports further development of the OVT73 model. After further characterization, ovine models may be poised to help bridge HD research from small animal to human models.

#### Other Animal Models

Relatively little work has been done with non-human primate and minipig models of HD, which have only recently been developed (8–10, 35–40). The literature search found no results for qEEG studies in these models and that behavioral phenotyping is currently ongoing in minipig models (35–37). A search for studies in non-human primates similarly yielded no results, and further investigation showed that non-human primate models are currently undergoing development and verification (9, 38–40). Both of these models are still under development and may have potential in the future to bridge the gap between rodents and humans.

### Human Studies

Research on the use of qEEG biomarkers in human subjects has included not only spectral frequency analysis as in animal models, but also other methods that take advantage of the larger size of the human brain and the greater number of electrodes to examine sources of electrical activity, regional differences in brain activity, and functional network complexity. These include source localization techniques such as low-resolution electromagnetic tomography (LORETA), topographic brain EEG mapping, and network and connectivity analyses (Table 2).

**Table 2:** **Literature review results for qEEG biomarkers in humans**.

Reference	Year	Measure	Subjects	Control	Pre-HD	HD	Electrophysiological Finding(s)	Clinical correlation(s)	CAG-repeat correlation	Other
Lazar et al. (41)	2015	EEG-PSG	82	36	38	8	5–7 Hz range (theta and alpha–theta border) relative power lower in pre-HD and early HD during REM relative to controls (*p* < 0.00125)	Relative power in 5–7 Hz range in HD during REM negatively correlated with disease burden score (DBS)	None	
							6–7 Hz range (theta and alpha–theta border) relative power lower in pre-HD and early HD during NREM relative to controls (*p* < 0.00125)			

Piano et al. (42)	2015	LORETA	46	23	0	23	Alpha power higher in HD during NREM relative to control (*p* < 0.01)	Delta LORETA power negatively correlated with Unified Huntington’s Disease Rating Scale (UHDRS) during wake	None	Includes a literature review table that goes back further than the one in this review
							Alpha power lower in HD during REM relative to control (*p* < 0.01)			
							Beta power lower in HD during NREM relative to control (*p* < 0.01)	Alpha, beta, and theta LORETA power positively correlated with UHDRS during NREM		
							Theta power lower in HD during NREM and REM relative to control (*p* < 0.01)			
							Delta power higher in HD during wake relative to control (*p* < 0.01)			

Ponomareva et al. (43)	2014	qEEG	58	29	29	0	8–9 Hz range (alpha) relative power lower in pre-HD relative to control (*p* < 0.05)	Alpha, theta, and delta relative power positively correlated with DBS in pre-HD	CAG repeat length positively correlated with delta and theta relative power, 4–5 and 5–6 Hz bin relative power	
							7–8 Hz range (alpha–theta border) relative power lower in pre-HD relative to control (*p* < 0.01)	Extreme positive correlation between the difference of relative power between 7–8 and 4–5 Hz bin and DBS	Negatively correlated with alpha relative power, 7–8, 9–10, and 10–11 Hz bin relative power
								Delta and theta relative power negatively correlated with FAS score	
								Alpha relative power positively correlated with FAS score	
								Extreme positive correlation between the difference of relative power between 7–8 and 4–5 Hz bin and FAS score	

Painold et al. (44)	2011	LORETA	110	55	0	55	Alpha LORETA power lower in HD relative to control (*p* < 0.05)	Alpha LORETA power positively correlated with Mini Mental State Examination (MMSE) score	None	Delta power observations were primarily in later stages of HD
							Beta LORETA power lower in HD relative to control (*p* < 0.05)			
							Theta LORETA power lower in HD relative to control (*p* < 0.05)	Theta and alpha LORETA power negatively correlated with UHDRS		
							Delta LORETA power higher in HD relative to control (*p* < 0.05)			

Painold et al. (49)	2010	EEG Mapping	110	55	0	55	Absolute alpha power lower in HD relative to control (*p* < 0.05)	Absolute alpha power positively correlated with MMSE score and negatively with UHDRS	None	
							Absolute beta power lower in HD relative to control (*p* < 0.05)	Absolute beta power positively correlated with MMSE and UHDRS scores		
							Absolute theta power higher in HD relative to control (*p* < 0.05)	Absolute delta power positively correlated with MMSE score and negatively with UHDRS		
							Absolute delta power higher in HD relative to control (*p* < 0.05)	Absolute theta power negatively correlated with MMSE and UHDRS scores		

Hunter et al. (45)	2010	qEEG	42	15	3	24	Absolute delta power higher in HD relative to control (*p* = 0.0000001)	Absolute alpha power negatively correlated with MMSE, digit symbol, and Stroop reading scores	Relative delta power AP gradient negatively correlated with CAG-repeat length	Absolute alpha power increase was only significant when medicated patients were included in analysis
							Relative alpha anterior–posterior (AP)-gradient lower in HD relative to control (*p* = 0.000004)	Relative alpha AP-gradient positively correlated with Burden Pathology Score (BPS) score and negatively with Total Functional Capacity (TFC) and Stroop reading scores		
							Relative delta AP-gradient lower in HD relative to control (*p* = 0.02)	Relative delta AP-gradient positively correlated with TFC score and negatively with BPS and Total Motor Symptoms scores		

Van der Hiele et al. (46)	2007	qEEG	29	13	16	0	Relative alpha power lower in Pre-HD relative to control (*p* = 0.003)	None	None	
							Absolute alpha power lower in Pre-HD relative to control (*p* = 0.013)			
							Theta power unchanged in Pre-HD relative to control (*p* = 0.36)			

De Tommaso et al. (47)	2003	qEEG	33	13	7	13	Absolute alpha power lower in HD relative to control (significance not reported)	None	None	No conclusions made regarding Pre-HD
							Absolute theta power higher in HD relative to control (significance not reported)			
							Absolute delta power higher in HD relative to control (significance not reported)			

*Base search terms included (EEG OR EEG Mapping OR qEEG) AND (Huntington’s Disease OR Huntington’s OR Huntington OR Huntington Disease). Additional terms included biomarker, preclinical, power spectral analysis, neural networks, and LORETA. Results were restricted to humans. Information noted includes recording modality used, the total number of subjects, the number of Huntington’s Disease (HD) subjects, the number of HDGEC/pre-HD subjects, the number of control subjects, any relevant qEEG-related findings, and correlations of those findings to clinical and genetic measures*.

De Tommaso et al. (47) applied three different methods of analysis (artificial neural network or ANN, Fisher linear discriminant, and likelihood ratio method) to examine qEEG data collected from HD subjects, premanifest HDGECs, and control subjects (47). Receiver-operating characteristic curve analysis showed that the ANN exhibited the strongest discrimination among groups of the three methods used. ANN analyses of HD subjects relative to controls showed a decrease in the absolute alpha power and an increase in both absolute theta and absolute delta power. None of these neurophysiologic measures correlated with any clinical or cognitive performance findings tested, including the Marsden and Quinn Chorea Severity Scale, Mini-Mental State Examination (MMSE), Wechsler Adult Intelligence Scale (WAIS) Full Scale IQ, WAIS Verbal IQ, WAIS Performance IQ, Verbal Scaled Score, and Block Design Scaled Score. The ANN neural scores for premanifest HDGECs did correlate with the predicted time to onset, indirectly suggesting an association between qEEG measures and the number of CAG repeats for those subjects.

Van der Hiele et al. (46) examined qEEG in control and premanifest HDGEC subjects in the context of a working memory task (46). They found a decrease in both relative and absolute alpha power compared to controls during the working memory task, but no differences were found during the resting state. No other statistically significant qEEG markers were found. The detected changes in alpha power during the working memory task had no clinical or pathophysiological correlates, including performance on the working memory task.

Hunter et al. (45) examined the anterior–posterior (AP) gradients of qEEG power in addition to global measures of relative and absolute qEEG power (45). This study replicated the global decrease in delta power in HD subjects relative to controls observed in prior reports (47, 48). This study further represented one of the first reports to examine regional qEEG patterns and found loss of the normal AP gradients of both alpha and delta relative power in HD subjects as compared to controls. Whereas relative alpha power normally shows a posterior-dominant distribution, loss of this posterior dominance was associated with greater disease burden and poorer performance as assessed by the Burden Pathology Score, Total Functional Capacity (TFC) score, and Stroop reading score measures. Regarding relative delta power, loss of the normal anterior-dominant distribution was correlated with lower TFC, higher disease burden (BPS), and higher total motor symptoms. Importantly, the delta relative AP gradient was significantly associated with the number of CAG repeats: loss of the relative delta AP gradient correlated with a higher number of CAG repeats. Furthermore, the relative alpha AP gradient was examined in three premanifest subjects and the gradient appeared to decrease in relation to estimated years until onset. Hunter et al. (45) was the first study to examine relative AP gradients in HD and the first to find a qEEG biomarker directly correlated to the number of CAG repeats.

Painold et al. (49) used qEEG mapping to examine spectral power, but with a larger sample size than previous qEEG HD studies in humans (49). They reported a decrease in both absolute alpha and beta power and an increase in both absolute theta and delta power relative to controls. Additionally, the frequency centroid of delta, theta, beta, and total power was decreased in HD relative to controls. Clinical correlates were also found: alpha and delta power positively correlated with MMSE scores and negatively with Unified Huntington’s Disease Rating Scale (UHDRS) scores, beta power positively correlated with both MMSE and UHDRS scores, and theta power negatively correlated with both MMSE and UHDRS scores. However, no correlates with CAG repeat length were reported. In a subsequent study, this group examined the same frequency bands using LORETA instead of EEG mapping as in the 2010 study (44). They discovered that theta, alpha, and beta LORETA spectral power decreased globally across early and late stages of HD, but increases in delta LORETA spectral power were only present in later stages of HD, and primarily in the right orbitofrontal cortex. The decrease in theta LORETA spectral power differs from Painold et al.’s 2010 study (44). However, theta and alpha power were still negatively correlated with UHDRS scores, and alpha power was positively correlated with MMSE scores. The source of the seemingly conflicting observations regarding theta power observations between Painold et al. (49) and Painold et al. (44) are unclear, as it appears that the same pool of subjects was used in both studies. There are, however, some differences in the regions where changes in theta power were observed; additionally, differences in analytical methods could explain the seeming discrepancy as LORETA utilizes a different transformation than EEG mapping to allow for source attribution of current density. Therefore, more global measures that are detected in EEG mapping are not necessarily observed in LORETA and vice-versa (42, 44, 49). Even so, both studies performed by Painold and colleagues identified qEEG measures that had significant association with clinical measures in HD.

Ponomareva et al. (43) approached the use of qEEG as a biomarker of HD in premanifest HDGECs and examined 1Hz frequency bins within the traditionally examined wave bands in premanifest and control subjects. It was also the first study to focus solely on pre-HD subjects using qEEG biomarkers (43). They found no significant differences between the relative power of pre-HD and control subjects when examining the broad frequency bands. However, relative power was found to decrease in pre-HD subjects in the 8–9 Hz sub-band and even more in the 7–8 Hz sub-band. Additionally, although no significant differences were found between the premanifest and control subjects in the broad bands, both delta and theta relative power demonstrated positive correlation with the number of CAG repeats within the pre-HD group, and relative alpha power demonstrated a negative correlation. The number of CAG repeats also correlated positively with the 4–5 and 5–6 Hz frequency bins, and negatively with the 10–11, 7–8, and 9–10 Hz bins. Highly significant positive correlation was observed between the Disease Burden Score (DBS) and the difference of relative power between the 7–8 and 4–5 Hz bins. The broad frequency bands were also found to correlate with clinical measures in premanifest subjects. Alpha, theta, and delta relative power in pre-HD subjects were all found to positively correlate with DBS. Word fluency (FAS) scores negatively correlated with delta and theta relative power, and positively with alpha relative power. The 8–9 Hz frequency bin relative power demonstrated positive correlation with FAS score, and the 4–5 and 5–6 Hz bins demonstrated negative correlation. The difference between the 7–8 and 4–5 Hz bin relative power again demonstrated highly significant positive correlation in FAS scores. Of note is that the investigators excluded frequency bands below 2 Hz, so that lower frequency delta activity was not examined. This affects the definition of the delta band as well as the total spectral power measured. Therefore, the relative power measurements found in Ponomareva’s study may differ from findings in other studies by virtue of the 2 Hz high-pass frequency cutoff. As noted, many of the frequency bands (both broad bands and 1 Hz bins) showed correlation with both the number of CAG repeats and clinical measures in pre-HD subjects. These EEG frequencies are of particular interest when identifying potential biomarkers. Further study in symptomatic HD subjects and HDGEC subjects is necessary to validate the use of these sub-bands as biomarkers.

Piano et al. (42) examined EEG in HD subjects using LORETA (42). While not as large as the studies from Painold and colleagues, Piano et al. provided valuable replication and examined the power spectrum characteristics in both wake and sleep states. Piano et al. found that during the wake state, delta LORETA power was observed to increase bilaterally in HD patients, though more strongly in the right hemisphere. During NREM sleep in HD subjects, alpha LORETA power was observed to increase bilaterally, theta LORETA power was observed to decrease bilaterally, and beta LORETA power was observed to decrease in the left hemisphere relative to controls. During REM sleep in HD subjects, alpha and theta LORETA power were both observed to decrease bilaterally compared to controls. EEG spectral LORETA power in Brodmann area 4 was associated with disease duration during wake and REM states and UHDRS score during NREM. Delta, theta, and left hemisphere beta LORETA power during the wake state were all positively associated with disease duration, while only right hemisphere delta was positively associated with UHDRS during wake. Theta, alpha, and beta LORETA power all correlated positively with UHDRS during NREM, and right hemisphere alpha LORETA power positively correlated with disease duration during NREM. During REM, alpha LORETA power again positively correlated with disease duration.

Lazar et al. (41) examined sleep and metabolic characteristics in premanifest and early HD subjects relative to controls and is the first study to examine sleep quality and metabolic markers in pre-HD subjects (41). No metabolic differences were found between the pre-HD and control subjects using *x*-ray absorptiometry, whole body indirect calorimetry, and blood sampling (for testosterone, cortisol, vitamin D, and leptin). However, differences were discovered in the principal component analysis of the qEEG data and sleep measures. Polysomnography measurements revealed that pre-HD and early HD subjects had objectively worse sleep quality than controls, and early HD subjects had worse quality of sleep compared to premanifest subjects. qEEG analysis showed a relative power increase in early HD subjects in the 5–7 Hz (theta/alpha–theta border) range compared to controls. The relative power of the 4–7 Hz range negatively correlated with DBS during REM sleep. Lazar et al. and Ponomareva et al. both examined smaller frequency bins, and they both found correlates in the alpha–theta border range (41, 43).

Many of the human studies identified potential biomarkers in similar frequency bands, although the specific changes within these bands is not entirely consistent. Four (45–47, 49) of the eight studies examined absolute power measures, three in HD subjects (45, 47, 49) and one in premanifest subjects (46). Global absolute alpha power was significantly lower in HD vs. controls in two of three HD studies (47, 49). Absolute alpha power was reported to decrease in premanifest subjects (49). Absolute delta power was found to increase in all three studies of HD subjects (45, 47, 49), but not in premanifest subjects (46). Absolute theta power was greater in HD relative to controls in two of the three studies (47, 49), but not in either the third (45) or in premanifest subjects (46). Absolute beta power was only observed to increase in one of the studies of HD subjects (49). Two of eight studies examined the relative power of 1 Hz frequency bins, particularly near the alpha–theta border (41, 43). One examined premanifest and early HD subjects relative to controls (43) and the other premanifest subjects relative to controls (41). Significantly, both found decreases in ranges near the alpha–theta border, though the specific bins that decreased were not consistent across the studies (41, 43). One of these studies excludes any signals under 2 Hz, making comparison of results across the two studies more difficult (43).

Using different analytic methods, two of the eight studies examined LORETA power (42, 44), derived from current-source density across Brodmann areas. However, Painold et al. examined LORETA during a vigilance state, while Piano et al. examined LORETA during NREM and REM sleep in addition to waking state. Delta LORETA power was higher in HD relative to controls during wake in both studies. While both studies have other results of interest, there was no overlap in other findings, as Piano et al. (42) found no other differences in HD relative to controls during wake, and Painold et al. (44) did not examine NREM and REM states.

## Discussion

The literature from preclinical and human studies suggest that qEEG measures constitute a reliable and reproducible biomarker to detect brain dysfunction in preclinical and human studies of the HD phenotype, as well as in HDGECs. Several potential biomarkers appear to replicate across human studies. One of the most replicable findings was an increase in absolute delta power, which has been reported frequently in HD subjects (42, 44, 45, 47, 49) although not in premanifest subjects (41, 43, 46). In premanifest HDGECs, the relative power of specific sub-bands has been observed to be decreased across multiple studies (41–44, 46, 47, 49). These sub-bands are at the border between alpha and theta frequencies (41, 43). These findings would suggest that in characterizing the process of phenoconversion in HDGECs, shifts in rhythmic oscillations between the alpha and theta frequency ranges may be particularly useful biomarkers. Hunter et al. (45) examined the AP gradients of relative qEEG power and found two more potentially useful biomarkers in the alpha and delta frequency bands (45). The effect of HD disease progression on rhythmic oscillatory activity in the delta, theta, and alpha bands constitutes a highly plausible disease biomarker for increasing mHTT burden: oscillations in this frequency range are modulated by corticothalamic circuits (50, 51), and in particular, by thalamic nuclei that are known to show structural changes with disease progression (52, 53). Different thalamic nuclei regulate rhythmic oscillations in anterior and posterior brain regions, which could explain why the AP gradient would shift during disease progression as different thalamic nuclei become involved in the illness. Other potential human biomarkers are in the higher frequency range, where beta absolute and LORETA power were observed to decrease in HD subjects (42, 44, 49). However, the decrease in beta power was not found in most studies examined (41, 43, 45–47). High-frequency power biomarkers have been correlated with clinical measures in at least one of the studies that examined them.

One of the most significant findings from the human biomarker literature is the correlation of specific qEEG variable with the number of CAG repeats: the relative delta AP gradient from Hunter et al. negatively correlated with CAG repeat length, the relative delta and theta power frequency bins from Ponomareva et al. positively correlated with the number of repeats, and the relative alpha power from Ponomareva et al. negatively correlated with the number of repeats (43, 45). Correlations with repeat length now have been reported in both symptomatic and premanifest subjects and are consistent with the fact that qEEG measures can be used to differentiate HDGECs from healthy controls before the onset of symptoms. This finding suggests that the production of mHTT has effects on neurophysiologic function even in advance of phenoconversion and offers the promise that qEEG biomarker might be useful for monitoring the efficacy of mHTT lowering therapies in clinical trials.

Inconsistencies or failure to replicate results across human biomarker studies may result from several factors. Studies frequently either do not report or statistically account for the medication status of their subjects. Psychotropic medication may affect qEEG power or frequency results, although Hunter and colleagues reported no significant effect of the most common medications. Other factors not commonly reported include age of onset, number of CAG repeats, and duration of symptoms. Such factors should be routinely examined in future studies.

For the purposes of treatment development, it would be useful for comparable qEEG biomarkers to be measureable in preclinical and clinical studies. The greatest body of preclinical work has been performed in rodent (primarily murine) models. There is a good consistency in rodent models in detecting changes in delta and theta power in HDGECs and affected animals. Most preclinical studies have reported decreased delta and theta power relative to WT animals, while human studies have reported increased power in the delta band but decreased power in the theta band relative to controls. Several factors may account for this seeming discrepancy in results. First, animal studies most commonly report absolute power (i.e., total energy output) differences, while the most robust differences in humans have been in relative power measures (i.e., proportion of energy in a band relative to the entire frequency spectrum). It is common in brain disease for the qEEG power to decrease in a slow wave band, but for the proportion of total power in that band to increase. As discussed above, human studies have reported shifts in power between the theta and alpha bands with phenoconversion, suggesting that relative power measures may be best suited to the purpose of biomarkers for mHTT lowering compounds. Future studies should compare absolute and relative power measures in murine models.

Second, the conditions in which qEEG recordings are performed in preclinical and human studies differ considerably. While human studies most commonly have been performed in the eyes-closed resting state or during a specific cognitive task, rodent studies have been recorded in a variety of waking conditions (resting or vigilance) or in the sleep state. Brain oscillatory patterns differ considerably across these conditions, and it is difficult to translate a specific waking state in mice to one in humans. It could be possible to compare sleep qEEG biomarkers in mice and humans, but little work has been done with qEEG during sleep in clinical populations.

Third, it is important to note that the frequency and location of oscillatory biomarkers is inherently different in rodent than human brain. Rodents do not have a posterior-dominant rhythm in the alpha frequency range as do humans, with broader theta frequency rhythms being more dominant. Rodents also have far less prefrontal cortex than do humans and a different pattern of mapping to thalamic nuclei that regulate rhythmicity. Therefore, there will be differences between species in both where and how qEEG biomarker differences can be detected. Owing to the small head size, rodent studies commonly record from only 1–2 cortical electrode locations in the anterior or central region. The largest qEEG biomarker differences between HD subjects or HDGECs and controls have been detected using topographic approaches to data analysis (i.e., regional brain mapping, AP gradients, and LORETA). It will be important for rodent studies of mHTT lowering biomarkers to measure qEEG in both anterior and posterior electrode locations, as well as in thalamic nuclei, in order to attempt to detect topographic effects. It also will be important to look for shifts in relative power oscillatory frequency rather than absolute power, given the fact that simple alpha and theta measurements do not translate across species.

Fourth, significant differences in high-frequency (beta and gamma) activity have been detected in rodent, but not in human studies. Several factors may account for these differences. Beta activity is easily measureable in both humans and animals, but in humans, it is more susceptible to recording artifact (e.g., muscle activity) as well as medication effects (i.e., benzodiazepines, which are commonly used by HD subjects to promote sleep). Gamma activity is a very low amplitude oscillation that is readily detected in animals with electrodes placed adjacent to or in the cortex, but is inherently difficult to measure in humans with extracranial scalp recordings that are susceptible to artifact from muscle tissue between the electrode and the cerebral sources. Beta frequency biomarkers may be detectable in clinical studies, but gamma frequency biomarkers may be better suited to rodent models, which can record gamma activity with greater reliability through the use of indwelling dural screw or cortical electrodes.

While qEEG biomarkers fulfill most of the criteria of fit-for-purpose biomarkers for mHTT lowering compounds, no studies yet have conclusively established a link between mHTT burden and a qEEG measure. And, while at least two studies have shown an association between a pharmacologic treatment intervention and normalization of a qEEG biomarker, no study has demonstrated sensitivity of a qEEG biomarker to an mHTT lowering intervention. To further establish the suitability of these biomarkers, longitudinal and cross-sectional research should be performed to replicate and extend previous findings. Replication would entail longitudinal studies examining the correlation of these biomarkers to both disease severity and number of CAG repeats in preclinical rodent models and human subjects. Extension of previous research would involve translation of preclinical biomarkers from rodents to humans by more precisely mapping the location and frequency bands that could establish equivalency of findings across species. A further extension also should be to suppress mHTT expression in rodents and observe the response of the biomarkers recorded in both cortical and subcortical areas known to be affected by disease progression. These biomarkers then could be examined in the context of other animal models such as sheep, minipigs, or non-human primates. This type of characterization would provide greater understanding of disease mechanism, validation of the biomarkers, and more precise definition of which biomarkers are fit-for-purpose for mHTT lowering studies in preclinical and human trials.

## Author Contributions

In terms of author contributions, MKL performed the review of the literature and wrote the first draft of the manuscript and AFL performed initial editing of the literature review and the manuscript. EJD, CC, AMH, AME-S, IAC, and MSL reviewed and edited the manuscript and contributed to the interpretation of the literature review.

## Conflict of Interest Statement

AFL discloses that, within the past 12 months, he has received research support from the National Institutes of Health, Neuronetics, Breast Cancer Foundation, Department of Defense, and Neurosigma. He has served as a consultant to NeoSync, Inc. He is Chief Scientific Officer of Brain Biomarker Analytics LLC (BBA). AFL owns stock options in NeoSync, Inc. and has equity interest in BBA. IAC has received research support from Covidien (formerly Aspect Medical Systems), Pfizer (formerly Wyeth Pharmaceuticals), and Eli Lilly and Company. He discloses that, within the past 3 years, he has received NIH grant support along with funding from Aspect Medical Systems/Covidien, NeoSync, and Neuronetics. He has been an advisor, consultant, or reviewer for Allergan, Neuronetics, NeuroSigma, NIH (ITVA), and the VA (DSMB). He has served on the Speaker’s Bureau or received honoraria from Neuronetics and NeuroSigma. His biomedical patents have been assigned to the Regents of the University of California. He has stock options in NeuroSigma, Inc. The remaining authors declare no conflict of interest.
